# Bilateral force control and coordination patterns across upper and lower limbs

**DOI:** 10.17179/excli2025-8672

**Published:** 2025-10-07

**Authors:** Tae Lee Lee, Nyeonju Kang

**Affiliations:** 1 Department of Human Movement Science, Incheon National University, Incheon, South Korea; 2 Division of Sport Science, Sport Science Institute, & Health Promotion Center, Incheon National University, Incheon, South Korea

**Keywords:** bilateral force control, interlimb coordination, isometric force control, hand-grip, ankle-dorsiflexion

## Abstract

Bilateral force control and coordination in upper and lower limbs are important functions for executing activities of daily living. Although upper and lower limbs may reveal distinct bilateral motor control patterns because of different motor networks involvements, no one has examined the possibility that upper and lower limbs reveal distinct bilateral force control and coordination patterns. This study investigated bilateral force control and coordination patterns between upper and lower limbs in healthy young adults. Thirty-two healthy young adults (mean±SD of age = 23.2±2.2 years; 16 Females) performed bilateral hand-grip and ankle-dorsiflexion force control tasks at 10 % and 40 % of maximal voluntary contraction. Bilateral force control performances were evaluated by calculating mean force, force symmetry, force accuracy, and force variability. To estimate bilateral force coordination, we used cross-correlation with time lag. Further, we examined the relationship between bilateral force control and coordination patterns of upper and lower limbs by conducting Pearson's correlation analysis. Bilateral maximal and mean forces of lower limbs were significantly less than those for upper limbs. At higher targeted force level, force accuracy and variability in lower limbs were significantly lower than those for upper limbs. More negative correlation coefficient values appeared in lower limbs as compared with upper limbs. Finally, bilateral force control performances in upper limbs were related to those in lower limbs although no significant correlation was observed for interlimb coordination patterns. These findings suggest that bilateral motor control and coordination patterns were different between upper and lower limbs although the level of bilateral upper and lower motor control capabilities was presumably influenced by shared motor control processes for each individual.

See also the graphical abstract(Fig. 1).

## Introduction

Bilateral force control in upper and lower extremities is a critical motor function for performing various activities of daily living (Kang and Cauraugh, 2015[21], Lee et al., 2024[27]). For example, bilateral hand-grip force control is frequently required for typing on a keyboard, driving a vehicle, and opening a water bottle (Kim et al., 2021[25]). Controlling ankle-dorsiflexion forces between feet is often used for walking, running, and ascending stairs (He et al., 2007[16]). Importantly, successful interlimb force control may be related to advanced sensorimotor processing to generate interactive motor commands from the central nervous system to the peripheral nervous systems (Enoka and Duchateau, 2017[8], Jin et al., 2019[19], Walsh et al., 2008[44]).

Potentially, neural activations patterns across key brain areas such as the primary motor cortex (M1), premotor cortex, supplementary motor area, posterior parietal cortex, basal ganglia, and cerebellum may be different between bilateral upper and lower movement executions (Archer et al., 2018[2], Rueda-Delgado et al., 2014[38], Walsh et al., 2008[44]). Specifically, a prior study reported that the arm areas of M1 may be connected to the premotor area, while the leg areas of M1 may be linked to spinal cord circuits so that these differences may reflect distinct functional roles in daily activities between upper and lower limbs (Volz et al., 2015[43]). In addition, posterior parts within the basal ganglia and cerebellum were activated for upper limb movements, whereas lower limb regions were associated with activation of anterior parts (Errante et al., 2023[9]). Further, neural communications between hemispheres through the corpus callosum contribute to executing improved interlimb force coordination (Kaminski et al., 2016[20], Long et al., 2016[29]). Interestingly, interhemispheric connectivity levels of corticospinal tract (CST) fibers from the leg areas of M1 were greater than those from the arm areas of M1 (Yeo and Jang, 2012[46]). These neural connections may facilitate more cortical activation between hemispheres during lower limb movements than upper limb movements (Kapreli et al., 2006[23], Noble et al., 2014[31]). Moreover, the properties of motor units may be different between upper and lower extremities (Dideriksen et al., 2012[7], Kim et al., 2001[26]). For instance, knee extensors with a greater number of motor units, a larger range of motor unit recruitment, and weaker synchronization patterns showed a reduction of force variability as compared with the first dorsal interosseus muscle (Kim et al., 2001[26], Tracy et al., 2007[41]). Potentially, different motor networks involved in upper and lower limb movements are associated with functional specificity in daily activities (Christou et al., 2003[3], Goel and Paloski, 2016[14]). Upper limbs often perform bilateral fine motor tasks, such as buttoning a shirt and playing instruments, whereas lower limbs are bilaterally used for gross and repetitive movements such as locomotion and postural control (Pérez, 2023[35], Volz et al., 2015[43]). Taken together, these findings raised a possibility that upper and lower limbs may reveal distinct bilateral force control and coordination patterns.

The purpose of this study was to determine whether bilateral force control and coordination patterns were different between upper and lower limbs in healthy young adults. We used bilateral hand-grip and ankle-dorsiflexion force control tasks at two submaximal targeted force levels (i.e., 10 and 40 % of maximal voluntary contraction: MVC) because bilateral force control can be affected by factors of neuromuscular organization such as force level (Hu et al., 2011[17]). Based on previous findings (Christou et al., 2003[3], Ohtaka and Fujiwara, 2019[32]), we hypothesized that bilateral force control performances and coordination in upper limbs would be better than those in lower limbs at lower targeted force level, whereas at higher targeted force level, lower limbs would show advanced bilateral force control performances and coordination than those in upper limbs.

## Methods

### Participants

Thirty-two healthy young adults (mean±standard deviation of age = 23.2±2.2 years; 16 females and 16 males) participated in this study. To identify the sample size, we performed a priori power analysis based on pilot data using G*Power software (version 3.1.9.6) (Faul et al., 2007[10]). The analysis confirmed that a minimum of 32 participants were required for a within-subjects design (power > 0.9 and alpha = 0.05). Participants were recruited from the community using flyers. The participants who met the inclusion criteria including age between 18 and 35 years, right-handed, and right-foot dominant, as measured by Edinburgh Handedness Questionnaire (Oldfield, 1971[33]) and ball-kicking tests (Sarabon et al., 2013[39]). We excluded participants who had musculoskeletal impairments across upper and lower limbs and cognitive deficits (Mini-Mental State Examination score < 28). Before starting the test, all participants read study protocols and signed an informed consent, approved by the Incheon National University's Institutional Review Board.

### Apparatus

**Figure 2:**
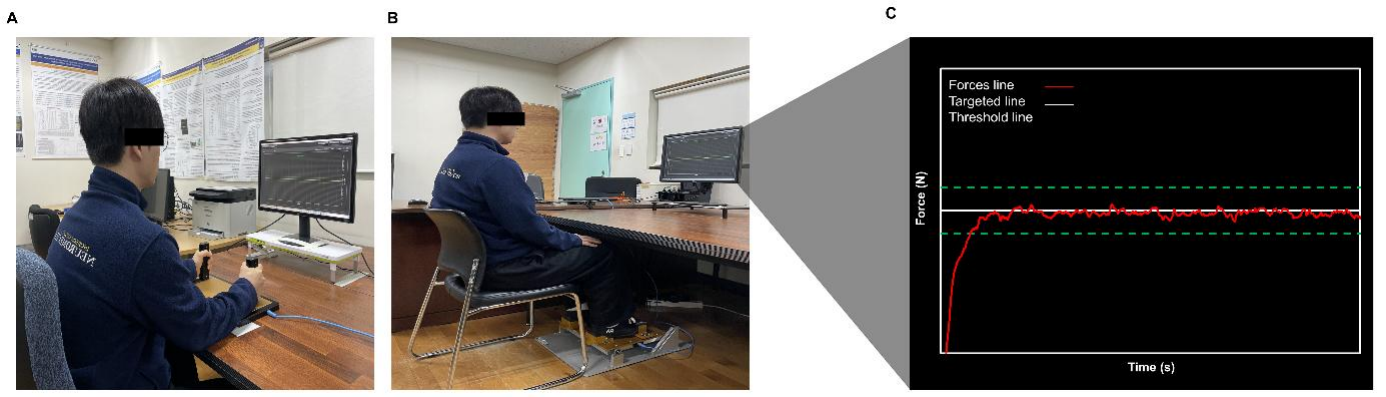
Experimental setup. (A) Bilateral isometric hand-grip force control task position. (B) Bilateral isometric ankle-dorsiflexion force control task position. (C) Visual information during force control tasks.

To perform bilateral isometric force control tasks with upper and lower limbs, we used a customized isometric hand-grip and ankle-dorsiflexion force control measurement devices (SEED TECH CO., Ltd., Bucheon, Republic of Korea), respectively. Participants were seated in front of a 54.6 cm LED monitor positioned 80 cm away. For bilateral hand-grip force control tasks, we instructed participants to place both arms on the table with comfortable positions (shoulder flexion 15-30° and elbow flexion 20-45°; Figure 2A(Fig. 2)), and participants grabbed the left and right handles embedded with two force transducers (Micro Load Cell-CZL635-3135, range = 330 lbs, Phidgets Inc., Calgary, Canada). During the task, bilateral isometric forces were generated by grasping left and right handles. To prevent unexpected force production from other part of the upper limbs (e.g., elbow, shoulder, and trunk), we instructed them to keep their forearms fixed on the table during the task.

For bilateral ankle-dorsiflexion force control tasks, participants were required to put their feet on the customized platform equipped with a force transducer for each size (Micro Load Cell-CZL635-3135, range = 330 lbs, Phidgets Inc., Calgary, Canada) in a comfortable position (knee flexion 90-100°, hip flexion 90-95°, and ankle dorsiflexion 90°; Figure 2B(Fig. 2)). To avoid unwanted movement caused by other lower limbs joints, we fastened the participant's metatarsals using metal straps modified to foot length and metatarsal height of each individual, and experimental shoes adjusted to the foot size of individuals were used. During the task, participants executed ankle-dorsiflexion movements with feet against the straps to generate isometric forces.

### Experimental procedures

Initially, participants conducted two consecutive maximum voluntary contraction (MVC) trials for upper and lower limbs (a duration of MVC trial = 5 s and 60 s rest between trials) to determine submaximal targeted force levels (i.e., 10 % and 40 % of MVC). We set four experimental blocks reflecting two limb conditions and two force levels, and randomly assigned the order of experimental blocks (1 block = 10 trials). For each force control trial, we provided three types of visual feedback on the screen (Figure 2C(Fig. 2)): (a) the red trajectory line = bilateral forces generated by left and right limbs, (b) the white horizontal line = targeted force level, and (c) two green dotted lines = ±10 % of threshold from targeted force level. The aim of force control trial was to generate and match bilateral isometric forces near a targeted force level for 20 s. We set a 30 s rest period between trials and a 60 s rest period between experimental conditions to minimize the impact of potential fatigue.

### Data analyses

Bidirectional fourth-order Butterworth filter with a 30 Hz of cutoff frequency was used for low-pass filter on all force data. To minimize potential initial adjustment and early termination effects, the middle 16 s of force data for each trial was analyzed after removing the first 3 s and the final 1 s of force data. All offline data analysis processes were conducted using the MATLAB programs (Math Works TM Inc., Natick, USA).

Bilateral force control performances were evaluated by calculating force production (i.e., MVC and mean force), symmetry, accuracy, and variability. Force symmetry was estimated by calculating a proportion of left mean force relative to right mean force for each trial so that values of force symmetry close to 1 indicate more symmetric force output between limbs, whereas values of force symmetry close to 0 denote more dependence on the dominant side of upper and lower limb (i.e., right hand and foot) (Lee et al., 2022[28]). To normalize potential effects of different force production between upper and lower limbs on force control patterns, we used relative root-mean-square error (rRMSE = RMSE / targeted force) for force accuracy and coefficient of variation ( %CV = standard deviation / mean force×100) for force variability. In addition, interlimb force coordination patterns were estimated by calculating cross-correlation coefficient and time-lag (Patel et al., 2019[34]). More positive values of cross-correlation coefficient (e.g., 0 < r ≤ 1) indicate in-phase coordination patterns, whereas more negative values (e.g., -1 < r ≤ 0) denote anti-phase coordination patterns. We calculated time-lag between the left and right limbs at the peak correlation to determine whether one force output preceded the other. More positive values of time-lag indicate that the force output of right limb leads the force output of left limb, whereas more native values denote that left limb force lead the right limb force.

### Statistical analyses

For statistical analyses, the Shapiro-Wilk test was conducted to confirm the normality of all dependent variables (Field, 2024[11]). An independent *t*-test was used for comparing MVC values between upper and lower limbs, and force control dependent variables were analyzed using two-way repeated measures ANOVA (Limb × Force Level; 2 × 2). Post-hoc analysis was performed using Bonferroni pairwise comparisons. Further, we conducted Pearson's correlation analyses to identify potential relationships between bilateral force control and interlimb coordination patterns in the upper and lower limbs. IBM SPSS Statistics version 28 (IBM Corp., Armonk, NY, USA) was applied for all statistical procedures and the alpha level was set at 0.05.

## Results

### Bilateral force control performances between upper and lower limbs

**Figure 3:**
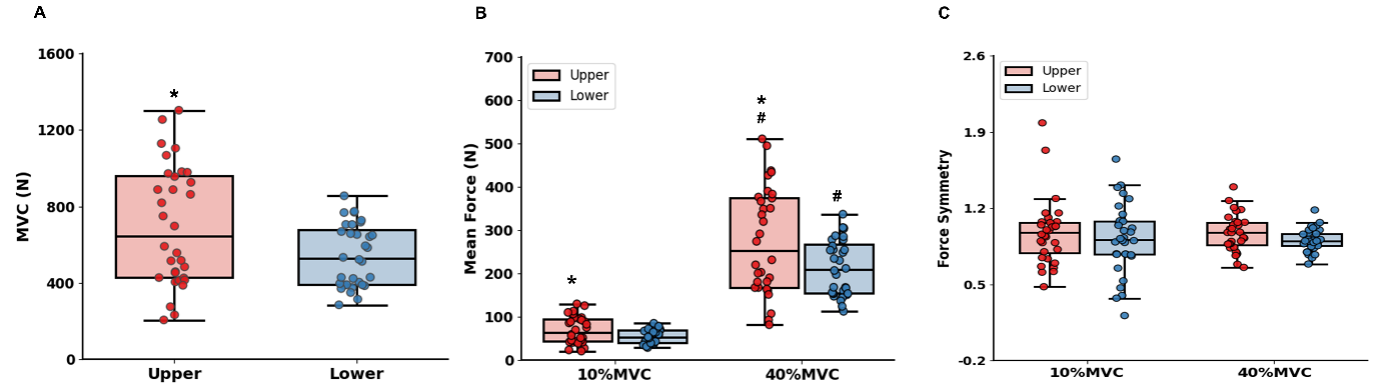
Bilateral force control performances findings. (A) Maximal voluntary contraction (MVC). (B) Mean force. (C) Force symmetry. *Asterisk* (*) shows a significant difference between the upper and lower limbs. *Number sign* (#) indicates a significant difference between 10 % and 40 % of MVC condition (*P* < 0.05).

An independent two-tailed *t*-test showed greater MVC values in bilateral hand-grip force control tasks than those in bilateral ankle-dorsiflexion force control tasks (*t_31_* = 2.497; *P* = 0.015; Figure 3A(Fig. 3)). For mean force during submaximal force control tasks, a two-way repeated ANOVA revealed a significant Limb × Force Level interaction [*F*(1, 31) = 23.090; *P* < 0.001; partial ƞ^2^=0.427; Figure 3B(Fig. 3)]. Post-hoc analyses demonstrated that mean bilateral hand-grip forces was higher than those in bilateral ankle-dorsiflexion forces for both submaximal targeted force levels. A two-way repeated ANOVA on the force symmetry showed no significant interaction [*F*(1, 31) = 0.019; *P* = 0.892; partial ƞ^2^=0.001; Figure 3C(Fig. 3)] and main effects: (a) Limb [*F*(1, 31) = 1.614; *P* = 0.213; partial ƞ^2^=0.049] and (b) Force Level [*F*(1, 31) = 0.047; *P* = 0.830; partial ƞ^2^=0.002].

**Figure 4:**
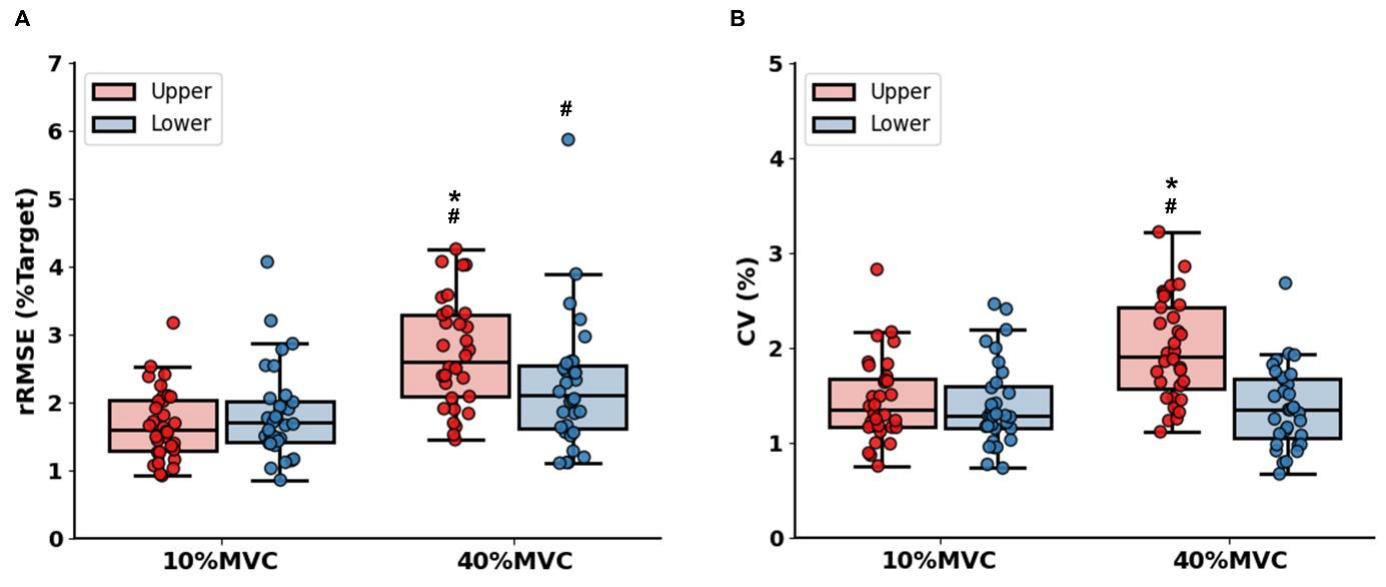
Bilateral force control performances findings. (A) Force accuracy (rRMSE). (B) Force variability (CV). *Asterisk* (*) shows a significant difference between the upper and lower limbs. *Number sign* (#) indicates a significant difference between 10 % and 40 % of MVC condition (*P* < 0.05).

A two-way repeated measures ANOVA on rRMSE revealed a significant Limb × Force Level interaction [*F*(1, 31) = 14.329; *P* < 0.001; partial ƞ^2^=0.316; Figure 4A(Fig. 4)]. Post-hoc analyses showed that rRMSE values in bilateral hand-grip force control tasks were higher than those in bilateral ankle-dorsiflexion force control tasks at 40 % of MVC (*P* = 0.006). For CV, the analysis showed a significant Limb × Force Level interaction [*F*(1, 31) = 22.986; *P* < 0.001; partial ƞ^2^=0.426; Figure 4B(Fig. 4)]. Specifically, CV values in bilateral hand-grip force control tasks were greater than those in bilateral ankle-dorsiflexion force control tasks at 40 % of MVC (*P* < 0.001).

### Bilateral force coordination patterns between upper and lower limbs

**Figure 5:**
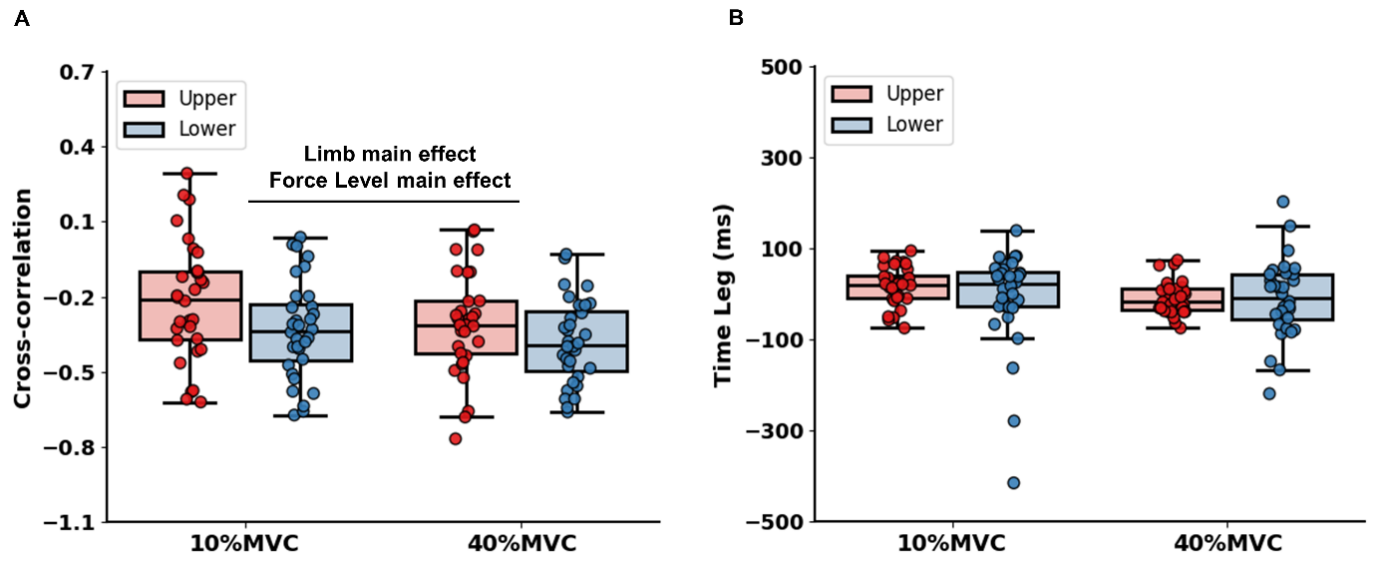
Bilateral force coordination findings. (A) Cross-correlation coefficient. (B) Time lag. *Asterisk* (*) shows a significant difference between the upper and lower limbs. *Number sign* (#) indicates a significant difference between 10 % and 40 % of MVC condition (*P* < 0.05).

A two-way repeated measures ANOVA on cross-correlation coefficient revealed a significant two main effects: (a) Limb [*F*(1, 31) = 5.260; *P* = 0.029; partial ƞ^2^=0.145; Figure 5A(Fig. 5)] and (b) Force Level [*F*(1, 31) = 6.097; *P* = 0.019; partial ƞ^2^=0.164]. Specifically, values of cross-coefficient in bilateral ankle-dorsiflexion force control tasks were more negative than those in bilateral hand-grip force control tasks collapsed across two targeted force levels. Further, values of cross-correlation coefficient were more negative from lower to higher targeted force levels collapsed across two limb conditions. The analysis on time-lag revealed no significant interaction [*F*(1, 31) = 1.296; *P* = 0.264; partial ƞ^2^=0.040; Figure 5B(Fig. 5)] and main effects: (a) Limb [*F*(1, 31) = 0.673; *P* = 0.418; partial ƞ^2^=0.021] and (b) Force Level [*F*(1, 31) = 1.581; *P* = 0.218; partial ƞ^2^=0.049].

### Correlations findings on bilateral force control between upper and lower limbs

**Figure 6:**
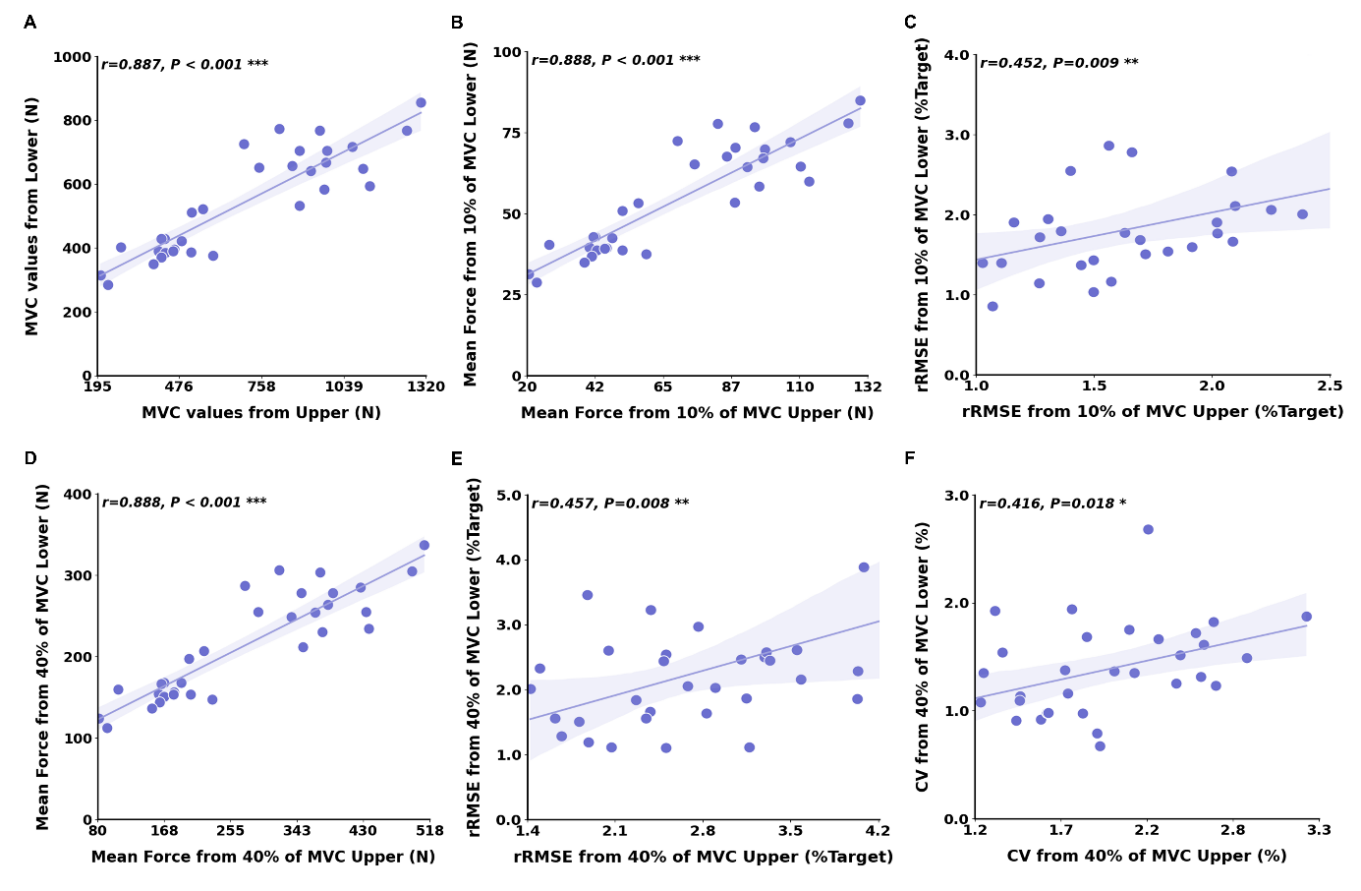
Correlation findings between upper and lower limbs. (A) MVC in upper versus lower limbs at 10 % of MVC. (B) Mean force in upper versus lower limbs at 10 % of MVC. (C) rRMSE in upper versus lower limbs at 10 % of MVC. (D) Mean force in upper versus lower limbs at 40 % of MVC. (E) rRMSE in upper versus lower limbs at 40 % of MVC. (F) CV in upper versus lower limbs at 40 % of MVC.

Pearson's correlation analyses revealed that bilateral force control performances at 10 % of MVC between hand-grip and ankle-dorsiflexion force control tasks were significantly correlated (Figure 6(Fig. 6); Table 1(Tab. 1)): MVC (*r* = 0.887; *P* < 0.001), mean force (*r* = 0.888; *P* < 0.001), and rRMSE (*r* = 0.452; *P* = 0.009). Moreover, bilateral force control performances at 40 % of MVC were significantly correlated between hand-grip and ankle-dorsiflexion force control tasks (Figure 6(Fig. 6); Table 1(Tab. 1)): mean force (*r* = 0.888; *P* < 0.001), rRMSE (*r* = 0.457; *P* = 0.008), and CV (*r* = 0.416; *P* = 0.018). However, interlimb force coordination patterns show no significant correlation between bilateral hand-grip and bilateral ankle-dorsiflexion force control tasks.

## Discussion

We examined bilateral force control and coordination patterns between upper and lower limbs in healthy young adults. Maximal and mean bilateral ankle-dorsiflexion forces were significantly less than those for bilateral hand-grip forces. Consistent with our hypothesis, relative force error and variability during bilateral ankle-dorsiflexion force control tasks were significantly lower than those during bilateral hand-grip force control tasks at higher targeted force level. Collapsed across two targeted levels, more negative interlimb coordination patterns appeared during bilateral ankle-dorsiflexion force control tasks as compared with bilateral hand-grip force control tasks. However, we found no significant differences in bilateral force accuracy and variability between upper and lower limbs at lower targeted force level. Finally, force accuracy and variability were correlated between bilateral hand-grip and ankle-dorsiflexion force control tasks although no significant correlation of interlimb coordination patterns was observed.

Lower maximal and mean bilateral ankle-dorsiflexion forces than bilateral hand-grip forces may be associated with different composition of muscle fibers (Reid et al., 2014[36]). Importantly, type Ⅰ fibers (i.e., slow-twitch fibers) contribute to maintaining lower levels of force outputs for a long-lasting period such as postural control and endurance performances, whereas type Ⅱ fibers (i.e., fast-twitch and fatigable fibers) are typically involved in producing explosive forces such as jumping and power-grip (Wilson et al., 2012[45]). For example, elbow flexors with more proportion of type Ⅱ fibers showed higher maximal forces than those in the first dorsal interosseus (Tracy et al., 2007[41]). Given that the tibialis anterior (TA) typically consists of lower proportion of type Ⅱ fibers (31.7 %) than forearm muscles (55.4 %) (Dahmane et al., 2005[5]), maximal and mean bilateral ankle-dorsiflexion forces may be less than those of bilateral hand-grip forces.

Despite no significant differences in force accuracy and variability between upper and lower limbs at lower targeted level, relative force error and variability during bilateral ankle-dorsiflexion force control tasks were lower than those for bilateral hand-grip force control tasks at higher targeted level. Considering higher correlations between force levels and force control performance, we used relative variables on force accuracy and variability. Nevertheless, increased relative accuracy and consistency of bilateral ankle-dorsiflexion forces at 40 % of MVC may be related to a range of submaximal forces frequently involved in performing lower limb activities of daily living (Kern et al., 2001[24]). Lower limb movement control such as walking, ascending stairs, and jumping were mostly associated with relatively higher-load (17-18 % of MVC) and long-lasting activities (Jacobs and Horak, 2007[18], Ohtaka and Fujiwara, 2019[32]), whereas upper limb movement control such as drawing and object manipulation required were lower-load tasks (6-8 % of MVC) (Arbib et al., 2009[1], Christou et al., 2003[3]). Moreover, given that greater proportion of type Ⅰ fibers correlated with the duration of muscle activity (Wilson et al., 2012[45]), sustaining and modulating bilateral ankle-dorsiflexion force outputs at a higher targeted level may be better than bilateral hand-grip force control with lower proportion of type Ⅰ fibers (Ohtaka and Fujiwara, 2019[32]).

Interlimb coordination patterns during bilateral ankle-dorsiflexion force control tasks were more negative than those for bilateral hand-grip force control tasks collapsed across two targeted force levels. Moreover, our correlation findings indicated no significant correlation of interlimb coordination between upper and lower limbs. Prior studies revealed that increased negative correlation patterns during bilateral force control tasks indicated error compensatory behaviors between left and right sides of limbs contributing to improved task performances (Hu et al., 2011[17], Lee et al., 2024[27]). Specifically, interhemispheric interactions between the primary motor cortices may increase error compensatory actions (i.e., anti-phase coordination patterns) and reduce mirror movements (i.e., in-phase coordination patterns) (Fling et al., 2013[12], Gooijers and Swinnen, 2014[15]). For example, older adults and patients with stroke who had lower interhemispheric connectivity between motor cortical regions often showed more deficits in interlimb force coordination (Fujiyama et al., 2012[13], Reis et al., 2008[37]). Transcallosal motor fibers, crucial for interhemispheric connections between two primary motor cortices, may be more involved in lower limb movements as compared with upper limb movements (Luft et al., 2002[30]) because many daily activities of lower limbs such as walking and running are associated with negative coordination patterns. Taken together, different interlimb coordination patterns between upper and lower limbs may be related to altered interhemispheric communication potentially affected by distinct motor skill specializations.

Interestingly, task performances significantly correlated between bilateral hand-grip and ankle-dorsiflexion force control tasks. These findings indicated that a performer may have shared motor control processes for bilateral force control capabilities across upper and lower limbs because of the homogeneous motor system (Tracy et al., 2007[41]). Specifically, the motor commands originating from the upper motor neurons are transmitted to muscles via the lower motor neurons to control movements of upper and lower limbs (Zayia and Tadi, 2020[47]). In fact, a prior study revealed that force variability during unilateral elbow flexion force control task was significantly related to those during unilateral knee extension force control task (Tracy et al., 2007[41]). Beyond the unilateral actions, our findings confirmed general bilateral motor control abilities between upper and lower limbs highlighting the interconnectedness of motor systems across different body regions in healthy individuals. Further research could explore whether similar patterns appeared in clinical populations or how task difficulty, muscle group involvement, and cognitive demand may influence these correlations.

Potentially, clinicians and physical therapists may consider the current findings when they apply bilateral movement rehabilitation programs. For example, given that no better bilateral force control at 10 % and 40 % of MVC appeared in the study, the intensity of bimanual movement rehabilitation protocols would be wide ranges for improving various daily activities including typing on a keyboard, manipulating objects, and carrying heavy items. For lower limbs, bimanual movement training may target improvements in lower force control such as walking, accelerator pedal control, and postural control. Moreover, considering the findings that older adults and neurological patients typically revealed impaired force control capabilities in upper limbs at lower and higher targeted force levels (Kang and Cauraugh, 2015[22], Vaillancourt and Newell, 2003[42]) and deficits in lower limbs at lower targeted force level (Skinner et al., 2019[40]), these rehabilitation strategies may be suggested for various populations.

Despite different bilateral force control and coordination patterns between upper and lower limbs, these findings should be carefully interpreted. First, given that we focused on hand-grip and ankle-dorsiflexion force control, the current findings may not appear in other comparisons including different parts of upper (e.g., wrists and fingers) versus lower limbs (e.g., knee and ankle-plantarflexion). Second, contrary to our hypothesis we found no better bilateral force accuracy and variability in upper limbs than lower limbs at 10 % of MVC. Potentially, given that ankle dorsiflexion is often involved in static and dynamic balance control requiring modulation of lower force outputs (Ohtaka and Fujiwara, 2019[32], Sarabon et al., 2013[39]), the motor system successfully performed bilateral ankle dorsiflexion force control tasks at lower targeted levels. Moreover, bilateral motor control patterns between upper and lower limbs may be affected by altered central and peripheral nervous systems so that future studies should determine whether distinct bilateral force control patterns between upper and lower limbs increase in patients with neurological diseases such as stroke and Parkinson's disease (Chung et al., 2023[4], Desrosiers et al., 2003[6]).

## Conclusion

In conclusion, the current study found that improved bilateral force control performances were observed in lower limbs as compared with upper limbs at higher targeted force level. Lower limb showed more negative coordination patterns than those for upper limbs collapsed across two targeted force levels. However, bilateral force control performances significantly correlated between upper and lower limbs. These findings suggest that bilateral motor control and coordination patterns may be different between upper and lower limbs across participants although the level of bilateral upper and lower motor control capabilities was presumably influenced by shared motor control processes for each individual. Future studies should investigate how altered bilateral force control performances and coordination patterns between upper and lower limbs are related to structural and functional changes in brain regions and motor neuron pools.

## Declaration

### Ethics statement

The studies involving human participants were approved by the Incheon National University Institutional Review Board. Prior to the study, all participants read and signed a written informed consent.

### Data availability statement

Correspondence and requests for materials should be addressed to NK.

### Authorship contribution statement

N.K. provided the idea of the research. T.L.L. conducted the experiments and data analysis. N.K. and T.L.L. contributed to data interpretation and manuscript drafts. Finally, N.K. reviewed the manuscript. All the authors read and approved the final manuscripts.

### Acknowledgments

The authors sincerely thank the study participants. This study is developed from Master Thesis of Tea Lee Lee completed at Incheon National University.

### Conflict of interest

The authors declare that they have no competing interests.

### Artificial Intelligence (AI) - Assisted Technology 

The authors declare that they have not used artificial intelligence for the writing of the manuscript and for the creation of graphics, figures, and tables.

### Funding

None.

## Figures and Tables

**Table 1:**
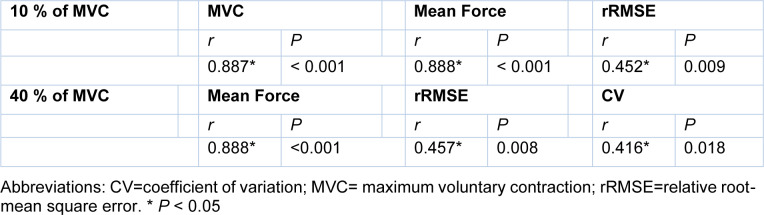
Correlation findings on bilateral force control between upper and lower limbs

**Figure 1:**
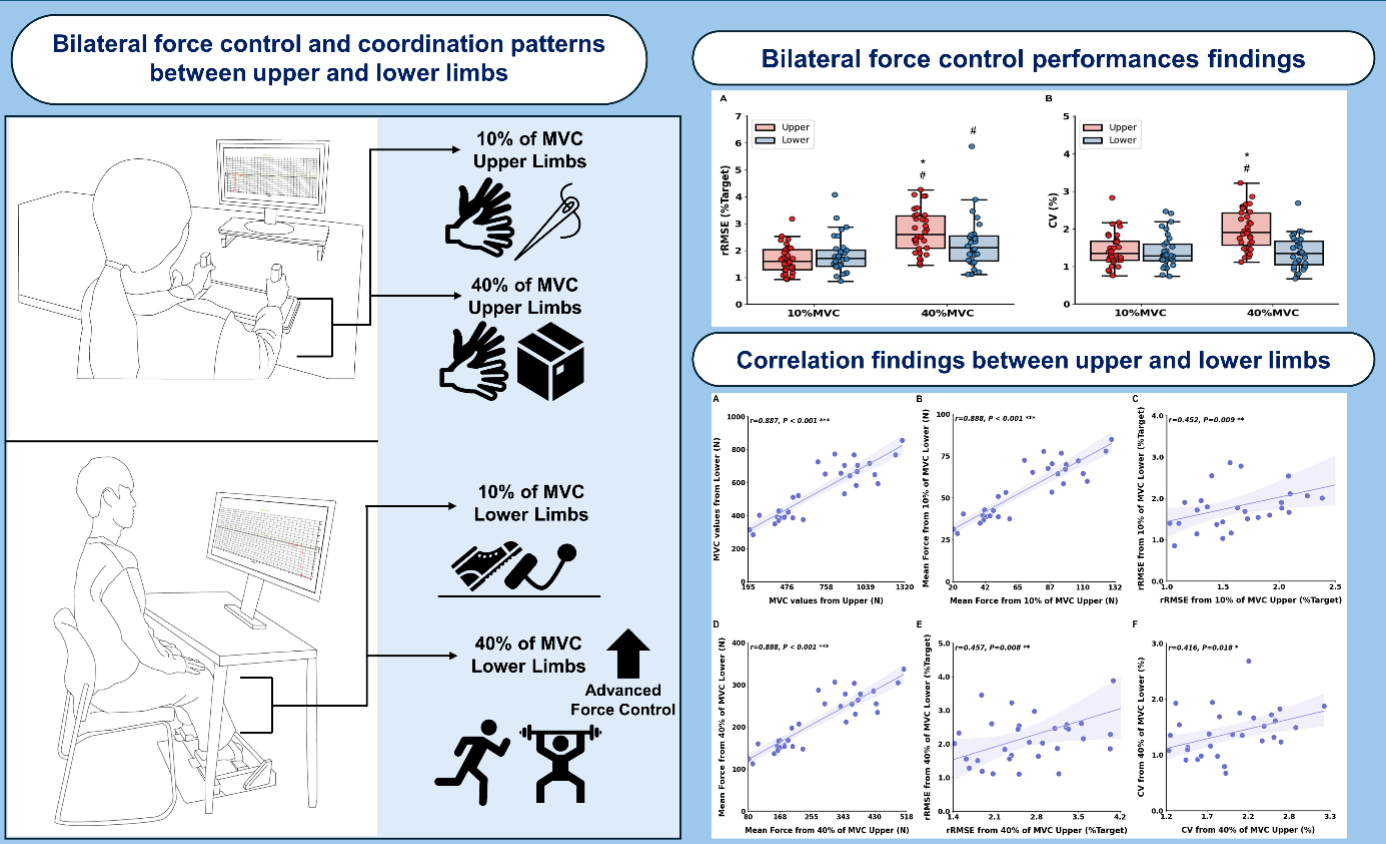
Graphical abstract
